# Naïve patients receiving TDF/FTC-EFV as 2 pills are more likely to modify regimen components than patients receiving a TDF/FTC/EFV single pill

**DOI:** 10.1186/1758-2652-13-S4-P122

**Published:** 2010-11-08

**Authors:** I Perez-Valero, N Martin, B San Jose Valiente, JI Bernardino-Serna, M Mora, F Gaya, J Gonzalez, FX Zamora, JM Pena, JR Arribas

**Affiliations:** 1 Hospital La Paz/Autónoma University School of Medicine. IdiPAZ, HIV Unit, Madrid, Spain; 2 Hospital La Paz/Autónoma University School of Medicine. IdiPAZ, Internal Medicine, Madrid, Spain; 3 Hospital La Paz/Autónoma University School of Medicine. IdiPAZ, Biostatistical Unit, Madrid, Spain; 4 Hospital La Paz/Autónoma University School of Medicine. IdiPAZ, Infectious Disease Unit, Madrid, Spain

## Purpose of the study

To estimate the short-term probability of treatment change in antiretroviral naïve patients receiving tenofovir (TDF), emtricitabine (FTC) and efavirenz (EFV) as a one (1p) or two pills (2p) regimen.

## Methods

We evaluated, by logistic regression and Cox proportional model analysis, factors associated to treatment modification during the first year of HAART in antiretroviral naïve patients from a single HIV unit in Madrid who started treatment with TDF, FTC and EFV as 1 p or 2p. For this analysis we censored patients who switched from 2p to 1p.

## Results

From Jan/06 to Dec/09, 136 patients started HAART with TDF, FTC & EFV as 1p (59, 42.8%) or 2p (79, 57.2%). Mean age: 38.5 (1p) and 38.6 (2p), 83.1% male (1p) and 75.3% (2p). Median CD4: 250 (1p) and 244 (2p), mean viral load (log): 4.53 (1p) and 4.48 (2p), HCV coinfected: 15.3% (1p) and 19.5% (2p). One-year probability of HAART modification was 14.7% (95%CI 9.7-21.6) globally, 20,78% (13.22-31.12) for 2p and 6.7% (2.67-16.18) for 1p. Proportions of patients with viral load <50 copies/mL after one year of follow up were 87.5% (1p) and 94.4% (2p). Reasons for HAART modification were toxicity (8.7%) and lack of efficacy (2.2%) or adherence (3.6%). HAART modification due to toxicity was more frequent (7.52%) with 2p (5 skin rashes, 2 SNC adverse events, 1 impairment of renal function and 1 osteopenia) than with 1p (2 skin rashes, 1.5%). Patients on 2p were changed to TDF/FTC + LPV/r (3), TDF/FTC + ATV/r (2), TDF/FTC + NVP (1), ABC/3TC + EFV (2), ABC/3TC + LPV/r (1), ABC/3TC + ATV/r (1), AZT/3TC/ABC + TDF (1) or stopped treatment (5). Patients on 1p switched to TDF/FTC + LPV/r (1), TDF/FTC + DRV/r (1) or stopped treatment (2). Multivariant logistic regression analysis showed that a 2p regimen [OR 5.0 (1.18-21.16)], prior AIDS-defining condition [4.09 (1.37-12.27)] and time (months) since HIV diagnosis [1.015 (1.006-1.025)] were significantly associated to HAART modification.

## Conclusions

Our study suggests that patients receiving TDF, FTC and EFV as 1p are more likely to maintain the regimen after one year than patients receiving the same regimen as 2p. Reasons for this difference might be related to a higher threshold for both clinicians and patients to change therapy even in the context of adverse events when patients are receiving TDF, FTC and EFV as a single pill.

